# 

**Published:** 1898-02

**Authors:** 


### NOTICES.

### Tenth Anniversary of the Odontographic Society of Chicago, Ills.

The Odontographic Society of Chicago, the largest dental association in the United States, excepting the American Dental Association, will celebrate its tenth anniversary, February 21st and 22d, 1898.

We shall spare no effort to make the event memorable in every respect, and to that end have planned a convocation which shall extend over two days and consist of clinics and scientific papers from men specially distinguished and representative in the science of dentistry.

You are cordially invited to attend and to present a paper, clinic or new dental appliance, as you see fit. The time is short for the extended preparations contemplated, therefore we are compelled to respectfully urge you to favor us with an early reply. Hoping to hear favorably from you, we are

Respectfully yours,
EXECUTIVE COMMITTEE,
C. E. Bently, *Chairman*,
100 STATE STREET, CHICAGO, ILLS.

